# A mixed methods analysis of the medication review intervention centered around the use of the ‘Systematic Tool to Reduce Inappropriate Prescribing’ Assistant (STRIPA) in Swiss primary care practices

**DOI:** 10.1186/s12913-024-10773-y

**Published:** 2024-03-18

**Authors:** Katharina Tabea Jungo, Michael J. Deml, Fabian Schalbetter, Jeanne Moor, Martin Feller, Renata Vidonscky Lüthold, Corlina Johanna Alida Huibers, Bastiaan Theodoor Gerard Marie Sallevelt, Michiel C Meulendijk, Marco Spruit, Matthias Schwenkglenks, Nicolas Rodondi, Sven Streit

**Affiliations:** 1 Institute of Primary Health Care (BIHAM), University of Bern, Bern, Switzerland; 2 Institute of Sociological Research, University of Geneva, Geneva, Switzerland; 3 Department of General Internal Medicine, Inselspital, Bern University Hospital, University of Bern, Bern, Switzerland; 4 Geriatrics, Department of Geriatric Medicine, University Medical Center Utrecht, Utrecht University, Utrecht, The Netherlands; 5 Department of Clinical Pharmacy, University Medical Center Utrecht, Utrecht, Utrecht, The Netherlands; 6 Public Health and Primary Care (PHEG), Leiden University Medical Center, Leiden University, Leiden, Netherlands; 7 Leiden Institute of Advanced Computer Science (LIACS), Faculty of Science, Leiden University, Leiden, Netherlands; 8 Department of Information and Computing Sciences, Utrecht University, Utrecht, Netherlands; 9 Health Economics Facility, Department of Public Health, University of Basel, Basel, Switzerland; 10 Institute of Pharmaceutical Medicine (ECPM), University of Basel, Basel, Switzerland; 11 Epidemiology, Biostatistics and Prevention Institute (EBPI), University of Zurich, Zurich, Switzerland; 12 Graduate School for Health Sciences, University of Bern, Bern, Switzerland; 13 Center for Healthcare Delivery Sciences (C4HDS), Division of Pharmacoepidemiology and Pharmacoeconomics, Department of Medicine, Brigham and Women’s Hospital and Harvard Medical School, Boston, United States

**Keywords:** Multimorbidity, Polypharmacy, Primary care, Medication optimization, Electronic clinical decision support system, Mixed methods research

## Abstract

**Background:**

Electronic clinical decision support systems (eCDSS), such as the ‘Systematic Tool to Reduce Inappropriate Prescribing’ Assistant (STRIPA), have become promising tools for assisting general practitioners (GPs) with conducting medication reviews in older adults. Little is known about how GPs perceive eCDSS-assisted recommendations for pharmacotherapy optimization. The aim of this study was to explore the implementation of a medication review intervention centered around STRIPA in the ‘Optimising PharmacoTherapy In the multimorbid elderly in primary CAre’ (OPTICA) trial.

**Methods:**

We used an explanatory mixed methods design combining quantitative and qualitative data. First, quantitative data about the acceptance and implementation of eCDSS-generated recommendations from GPs (*n* = 21) and their patients (*n* = 160) in the OPTICA intervention group were collected. Then, semi-structured qualitative interviews were conducted with GPs from the OPTICA intervention group (*n* = 8), and interview data were analyzed through thematic analysis.

**Results:**

In quantitative findings, GPs reported averages of 13 min spent per patient preparing the eCDSS, 10 min performing medication reviews, and 5 min discussing prescribing recommendations with patients. On average, out of the mean generated 3.7 recommendations (SD=1.8). One recommendation to stop or start a medication was reported to be implemented per patient in the intervention group (SD=1.2). Overall, GPs found the STRIPA useful and acceptable. They particularly appreciated its ability to generate recommendations based on large amounts of patient information. During qualitative interviews, GPs reported the main reasons for limited implementation of STRIPA were related to problems with data sourcing (e.g., incomplete data imports), preparation of the eCDSS (e.g., time expenditure for updating and adapting information), its functionality (e.g., technical problems downloading PDF recommendation reports), and appropriateness of recommendations.

**Conclusions:**

Qualitative findings help explain the relatively low implementation of recommendations demonstrated by quantitative findings, but also show GPs’ overall acceptance of STRIPA. Our results provide crucial insights for adapting STRIPA to make it more suitable for regular use in future primary care settings (e.g., necessity to improve data imports).

**Trial registration:**

Clinicaltrials.gov NCT03724539, date of first registration: 29/10/2018.

**Supplementary Information:**

The online version contains supplementary material available at 10.1186/s12913-024-10773-y.

## Background

Globally the proportion of adults with multimorbidity has increased in past decades [1, 2]. More than 50% of older adults aged ≥ 65 years have several chronic conditions [3]. The coexistence of ≥ 2 chronic conditions is commonly referred to as multimorbidity [4]. Multimorbidity is usually accompanied by polypharmacy, which can be defined as the concurrent, regular intake of ≥ 5 medications [5]. The higher the number of medications used, the more likely older adults are to have potentially inappropriate polypharmacy, which not only consists of the use of inappropriate medications, but also prescribing omissions [6–10]. The use of potentially inappropriate medications, highly prevalent in older adults with multimorbidity and polypharmacy [11], is associated with an increased risk of adverse drug events, falls, and cognitive decline in older adults [12–16]. This in turn is associated with increased health services use, such as hospitalizations or emergency department visits, and higher healthcare costs. Hence, optimizing medication use of older adults with multimorbidity and polypharmacy is a crucial task.

However, performing medication reviews is time-consuming and can be challenging, especially in a context in which time allocated to treating individual patients is short, as is commonly the case in primary care settings, and large amounts of patient information need to be processed (e.g., medications, diagnoses, lab values, patient preferences). Considering new possibilities available through the digital revolution, electronic clinical decision support systems (eCDSS) can be a useful tool for supporting healthcare professionals, when performing medication reviews. eCDSS are software-based tools, able of managing large amounts of data and designed to be a direct aid to clinical decision making [17]. They are capable of matching information, such as evidence-based clinical recommendations (e.g., guidelines), with patient information and can thereby generate patient-specific recommendations.

One such eCDSS is the ‘Systematic Tool to Reduce Inappropriate Prescribing’ Assistant (STRIPA). It is based on the algorithms of the ‘Screening Tool to Alert doctors to Right Treatment’ (START) and ‘Screening Tool of Older Person’s Prescriptions’ (STOPP) version 2 [18]. The STOPP/START criteria are the most widely used and extensively studied explicit screening tool to detect potentially inappropriate prescribing in older patients in Europe [19, 20]. While the STOPP criteria highlight situations of potentially inappropriate medication use (e.g., overprescribing, drug-drug interactions, drug-disease interactions, incorrect dosages), the START criteria indicate potential prescribing omissions. The STRIPA generates patient-specific recommendations, based on the STOPP and START criteria, by considering medication lists, diagnoses, and selected lab values [21]. It is thus a promising tool for optimizing pharmacotherapy in older adults and has been tested in two clinical trials to determine if its use can improve clinical outcomes (e.g., European multicenter hospital-based OPERAM trial in Switzerland, the Netherlands, Belgium and Ireland [22, 23], OPTICA trial in Swiss primary care settings [24–26].

The use of eCDSS has been shown to be beneficial for certain medication-related outcomes, such as reductions of medication errors, improvements in prescribing quality and decreases in the use of potentially inappropriate medications, which in turn leads to increased medication safety [27–29]. However, the evidence supporting the use of eCDSS largely focuses on hospital settings and results are mixed for primary care settings [30]. More specifically, current evidence shows high variability in the effectiveness and implementation of such tools in primary care settings and reports implementation challenges (e.g., time-consuming data entry, alert fatigue) [31–34]. Such documented problems related to implementing these tools can be hypothesized to have negatively influenced the impact of their use. Consequently, studying eCDSS implementation in primary care settings is crucial, as this will influence the future development of effective implementation strategies. In this context, the present study aimed to explore the implementation of the medication review intervention centered on the use of the STRIPA during the ‘Optimising PharmacoTherapy In the multimorbid elderly in primary CAre’ (OPTICA) trial conducted in Swiss primary care settings by using an explanatory mixed-methods approach. Our goal was to analyze the number of prescribing recommendations generated and implemented, the time expenditure for performing the intervention, and the key themes emerging from interviewing general practitioners (GPs) about their use of the intervention.

## Methods

**Fig. 1:**
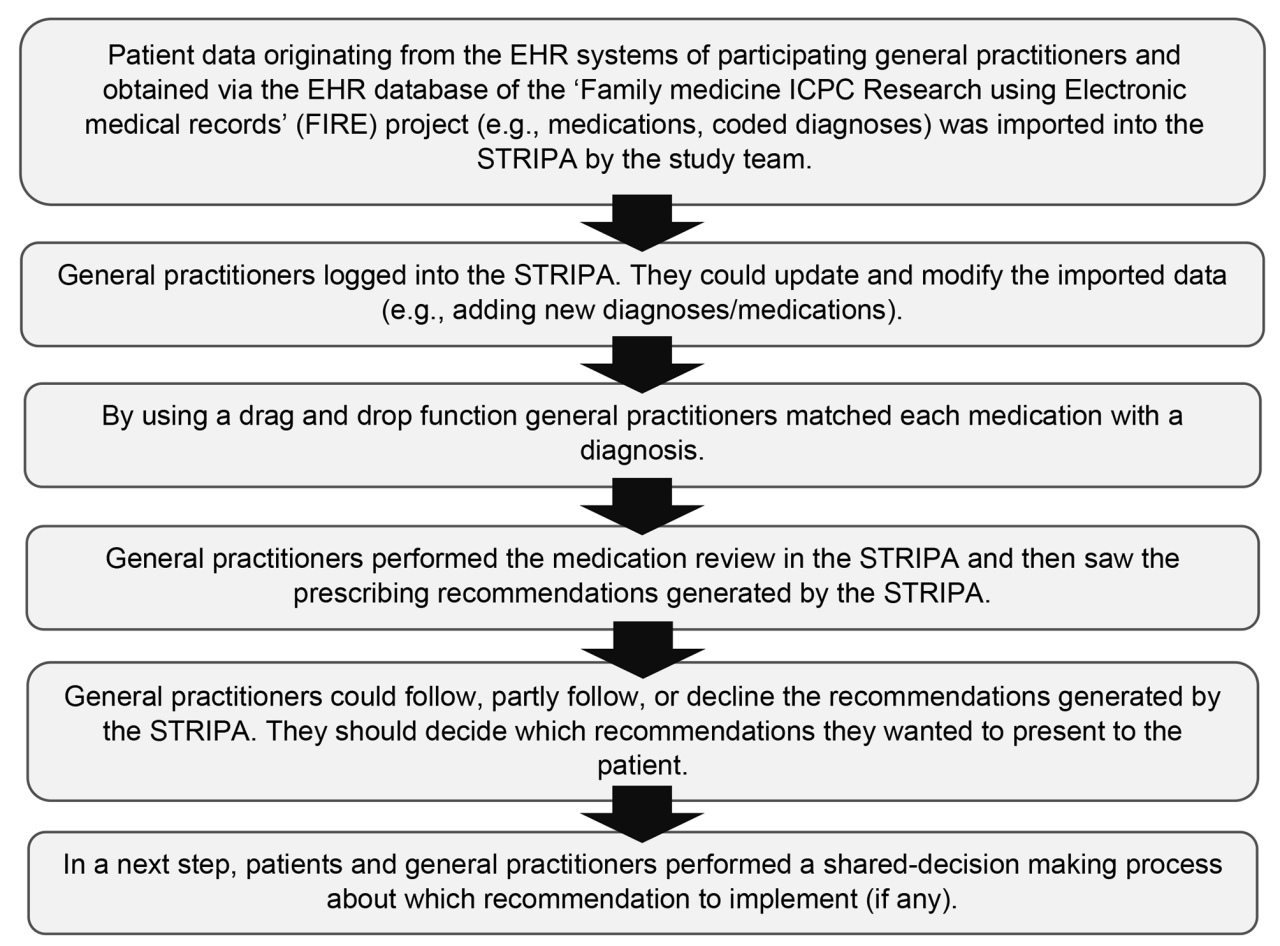
Schema of the six steps of the OPTICA study intervention using the ‘Systematic Tool to Reduce Inappropriate Prescribing’ (STRIP) assistant. Adapted from: Jungo et al. [24]

This research was embedded in the OPTICA trial [26], a cluster randomized controlled trial in Swiss primary care practices conducted by an interdisciplinary and interprofessional team (e.g., GPs, epidemiologists, etc.). The main goal of this trial was to investigate whether the use of a structured medication review intervention centered around the use of an eCDSS, namely the ‘Systematic Tool to Reduce Inappropriate Prescribing’ Assistant (STRIPA), helps to improve the medication appropriateness and reduce prescribing omissions in older multimorbid adults with polypharmacy compared to a medication discussion between GPs and patients [24–26]. The details of the trial protocol and the baseline characteristics of study participants have previously been reported [24, 25]. Fig 1 provides an overview of the different steps of the intervention. In addition to detecting potential overuse, underuse, and misuse of drugs, STRIPA generated prescribing recommendations to prevent drug-drug interactions and inappropriate dosages, by combining both implicit and explicitly tools to improve appropriate prescribing [21]. The version of the STRIPA used for the OPTICA trial had been adapted for use in primary care settings from the STRIPA version used in the OPERAM trial conducted in four European countries, in which the medication review intervention was done during hospitalization [22, 23, 35]. The data on medications, coded diagnoses, laboratory values, and vital signs originating from the electronic health records (EHR) of participating GPs and their patients were imported into the STRIPA by the study team after they were obtained from the ‘Family Medicine ICPC-Research using Electronic Medical Records’ (FIRE) EHR database [36]. Trial participants were ≥ 65 years old, had ≥ 3 chronic conditions, regularly used ≥ 5 medications and were followed-up for 12 months. In the intervention arm GPs used the STRIPA to perform a medication review and engaged in shared-decision making with patients. Trial results were inconclusive on whether the medication review intervention centered around the use of an eCDSS led to an improvement in medication appropriateness or a reduction in prescribing omissions at 12 months compared to a medication discussion in line with usual care (without medication review). Nevertheless, the intervention was safely delivered without causing any harm to patients and led to the implementation of several prescribing recommendations [26].

### Study design

In this sub-study, we used a mixed methods design in which we combined information collected from participating GPs on the prescribing recommendations generated and implemented and semi-structured interviews with GPs from the OPTICA intervention group. In an explanatory approach, we first collected quantitative data, which we sought to subsequently further explain and understand through qualitative methods [37]. We reported the findings of this study according to the CRISP statement [38].

### Participants

In both the quantitative and qualitative part of the research project, the study participants were the GPs who were randomly assigned to the intervention arm of the OPTICA trial (*n* = 21).

### Data collection

#### Quantitative component

Since during the trial all GPs from the OPTICA intervention group had access to the medication review intervention centered around STRIPA and were asked to perform it with their recruited patients, we invited all of them to report information on the use of the intervention in the REDCap study database. This covered the number of generated and the implemented prescribing recommendations, which are relevant outcomes to study the implementation of a medication review intervention. In addition, GPs had the option of providing free text responses on why they did not implement any prescribing recommendations. KTJ verified the entries in REDCap and completed them with information available in STRIPA. The following variables were collected for each recommendation generated: name of the recommendation, type of the recommendation, whether the recommendation was presented to the patient, and (if applicable) whether the recommendation was implemented. Furthermore, GPs directly reported the time used to prepare and conduct the medication review as well as the time spent on the shared decision-making with the patient. Quantitative data were collected between May 2019 and February 2020.

#### Qualitative component

We performed semi-structured interviews with a purposive sample of intervention group GPs who had been included in the OPTICA study. Interviews were conducted by FS in Swiss German and transcribed verbatim to High German. The interview guide included questions related to GPs’ attitudes towards treating older adults with multimorbidity and polypharmacy, the conduct of the medication review intervention tested during the OPTICA trial, and GPs’ general attitudes towards the use of eCDSS for optimizing prescribing practices (Appendix 1 in the Supporting Material). Preliminary quantitative data were used to inform the interview guide (e.g., quantitative findings about the implementation of prescribing recommendations and the use of the eCDSS, such as “We saw that it took around 40 minutes to prepare and perform the intervention. How does that compare to your experience during the trial when conducting the intervention?”), so that GPs could provide information on their perspective. Interviews were audio-recorded and transcribed into text for analysis. Interviews were conducted between October 2019 and February 2020.

### Data analysis

#### Quantitative component

We described participant baseline characteristics and performed descriptive analyses. We calculated the total number of recommendations generated per study participant in the OPTICA intervention arm. We then calculated the number of recommendations physicians reported to have discussed with patients and the number implemented after shared decision-making. In addition, we calculated the average time spent on preparing and conducting medication reviews and the average time of shared decision-making consultations. Since some variables were non-normally distributed (visual test), we present mean (standard deviation) and median (interquartile range). We performed all analyses with Stata 15.1 (StataCorp, College Station, TX, USA) [39].

#### Qualitative component

We analyzed the qualitative data with *thematic analysis*, which is a commonly used tool to identify and analyze patterns in qualitative data [40]. We used a mix of deductive and inductive coding, with deductive coding allowing us to expand on specific findings from the quantitative results and inductive coding allowing us to interpret any surprising findings we had not expected. Three of the investigators (KTJ, MJD, FS) contributed to the identification of themes. Consensus was reached by discussing the themes that were independently identified. In addition, we used the Framework method by Gale et al. to structure our analyses [41]. We used the software TamsAnalyzer to code and organize qualitative data into meaningful themes [42].

## Results

### Baseline characteristics

There were a total of 21 GPs and 160 of their patients in the intervention group. Table 1 provides baseline characteristics of the GPs and patients in the OPTICA intervention group.

**Table 1:** Baseline characteristics of general practitioners and patients in the OPTICA intervention group

**General practitioners (** ***n*** ** = 21)**	
Practice location	
Rural (%)	9 (43%)
Urban/suburban (%)	12 (57%)
Age in years, mean [SD], median [IQR]	50 [9], 51 [44 to 58]
Female (%)	3 (14%)
Work experience as general practitioner (GP) in years, mean [SD], median [IQR]	14 [9], 12 [8 to 22]
Average number of consultations per workday, mean [SD], median [IQR]	23 [5], 25 [20 to 25]
Practice form	
Individual practice (%)	2 (10%)
Group practice (%)	19 (90%)
Self-dispensation of medications in GP office	
Yes (%)	7 (33)
No (%)	14 (67)
Drug-drug interaction checker available as standard of care in her systems	
Yes (%)	1 (5)
No (%)	20 (95)
**Patients (n=160)**	
Age in years, mean [SD], median [IQR]	78 [7], 77 [73 to 83]
Female (%)	71 (44%)
Highest education level	
Less than mandatory schooling (%)	3 (2%)
Mandatory schooling (%)	56 (35%)
High school degree or apprenticeship (%)	75 (47%)
University or equivalent (%)	22 (14%)
Other (%)	2 (1%)
Number of long-term medications at baseline, mean [SD], median [IQR]	7 [4], 7 [4 to 10]
Number of chronic conditions at baseline, mean [SD], median [IQR]	7 [5], 6 [4 to 9]

IQR = interquartile range. │Table adapted from: Jungo et al. [26]

Table 2 shows the expenditure of time, per patient, for the preparation of the STRIPA, the conduct of the medication review intervention, as well as the duration of discussion with the patient. We observed that the drag/drop function to assign drugs to medical conditions in the STRIPA had been used for 133 out of the 160 patients in the intervention group, by 20 of the 21 GPs. GPs in the intervention group conducted a mean of 6 medication reviews (median = 7). For the 133 patients, a minimum of one prescribing recommendation had been generated for 130 patients (97.7%). A total of 704 prescribing recommendations had been generated for patients in the intervention group [26]. For the 133 patients, an average of 3.7 STOPP/START recommendations (SD 1.8, range: 0–11, median = 3, IQR = 2–5) was generated by STRIPA per patient. The mean number of STOPP recommendations generated by STRIPA was 2.3 (SD 1.3, range: 0–7, median = 2, IQR = 1–3) per patient and the mean number of generated START recommendations was 1.3 (SD 1.2, range: 0–6, median = 1, IQR = 1–2). For 53 patients in the intervention group, 10 of the GPs provided information on the implementation of prescribing recommendations. For 31 out of the 53 patients (58.5%) at least one prescribing recommendation was reported to have been implemented. On average, 1 recommendation to stop or start a medication was reported as implemented per patient (SD = 1.2, median = 1, IQR = 0–2). The most common reasons why GPs reported not implementing the prescribing recommendations were: beliefs that current prescriptions were beneficial for patients, recommendations were not suitable for patients, and bad experiences with previous medication changes.

**Table 2:** Information about expenditure of time for preparation and use of the STRIPA and the discussion of prescribing recommendations with the patients

GPs:*n* = 10 / Patients:*n* = 76	Minutes	
	Median (IQR)	Mean (SD)
Preparation time	12.5 (25)	25 (34)
Medication review using the STRIPA	10 (10)	14 (16)
Discussion durations with patients	5 (5)	8 (8)

Information on the expenditure of time was reported by 10 GPs from the intervention group about 76 of their patients

### Quantitative findings

#### Qualitative findings

Overall, semi-structured interviews were conducted with 8 of the 21 GPs randomized to the intervention group. The qualitative results allowed us to focus more specifically on GP perspectives on, and experiences with, STRIPA and to support our understanding of the limited implementation documented in the quantitative findings (e.g., significant time expenditure and limited implementation of prescribing recommendations). GPs generally appreciated the fact that the STRIPA was able to manage a large amount of data and to generate different types of prescribing recommendations, such as discontinuing or initiating medications. Despite this general appreciation, we identified the following themes as being barriers for GPs for STRIPA use: length of time for STRIPA preparation, problems with data sources, and poor data quality, sub-optimal functionality, limited recommendation practicability, and problems related to the implementation of recommendations.

#### Preparation

Most GPs mentioned that the coding of diagnoses (to ICPC-2) in their EHR systems was a time-consuming and cumbersome task because most did not routinely use it prior to the beginning of the trial. GPs found the expenditure of time to prepare the STRIPA, including the coding of diagnoses, too high. For instance, one GP (male, 57 years) stated, “*I was a little overwhelmed by the administrative burden*”. It also became clear that the lengthy time expenditure involved in preparing the STRIPA would be a limiting factor for the tool’s future use: *“if time expenditure remains that high, the STRIPA has no chance of being used in clinical practice*” (GP, male, 44 years). It was also stated that this long preparation time would not have made it possible for GPs to use the tool during the consultations with patients present.

#### Data import

Another major theme involved sub-optimal completeness of data imported from EHR systems to web-based STRIPA, which created additional work for GPs. Problems with data imports were multifaceted. First, not all information needed for STRIPA use was systematically captured in EHR systems and fully exported to the FIRE project database. For instance, this concerned unstructured information in text fields and lab values for which the FIRE team did not yet standardize imports into their database. Second, there was a time lag of up to a couple of weeks, because as explained above, data were transferred via data exports from the physicians' EHR systems to the FIRE project database and then back to the STRIPA. This required data to be updated and verified once they were in the STRIPA. Overall, GPs expressed that this time-consuming data updating and correcting was a limiting factor for future use of the STRIPA: *“I had to capture quite a lot of information by hand, and that is of course terribly tedious and time-consuming and thus not suitable for daily practice*” (GP, male, 44 years^1^). Some GPs mentioned how they would have appreciated an automated data transfer from the EHR system used in their GP office to the STRIPA, as this would have facilitated their use of the tool.

#### Functions and features

Overall, GPs reported to be satisfied with the functions and features of the STRIPA. For instance, GPs appreciated STRIPA’s ability to incorporate a wide variety of values into analysis (i.e., different lab values, medication lists, diagnoses, vital signs), which they would not have been able to do manually. Further, GPs described how they appreciated the varied types of prescribing recommendations, since this highlighted different types of prescribing-related problems. However, not all GPs thought the tool was intuitive to use. Further, some GPs reported technical problems when using the tool (e.g., long buffering when loading a new page or the next step of the analyses, problems with downloading PDF reports). GPs also noted a learning effect (e.g., after getting to know the tool, GPs were able to perform the subsequent reviews faster).

#### GPs’ perceptions of the suitability and practicability of recommendations

GPs reported being satisfied with the overall quality of recommendations. However, GPs emphasized that recommendations were not always suitable, practicable or clinically relevant. First, due to the above-mentioned problems with data imports, recommendations were sometimes not applicable for patients. For example, there may have been valid reasons why certain medications were prescribed at certain doses, and these reasons were not captured in the STRIPA. Second, recommendations were sometimes not suitable because of the seasonality of recommendation (i.e., influenza vaccine: most GPs used the STRIPA in spring 2019, which did not correspond to the influenza vaccination season). Furthermore, in the EHR systems GPs usually did not list the influenza vaccine to the regular medications used by their patients, which is why the recommendation to vaccinate appeared, irrespective of whether the patient had been vaccinated in the past fall. Third, in some cases, the STRIPA could not use all information provided (e.g., it did not capture that some medications had several active ingredients). In some instances, GPs reported not implementing certain recommendations as they did not believe that these recommendations would change patient health-status or well-being.

Further, some recommendations were perceived as too basic and therefore not useful for experienced GPs. One GP put it like this: “*Some of the information provided is not necessary for an experienced general practitioner*” (GP, male, 44 years). In some instances, the STRIPA generated prescribing recommendations that were already known to the GPs but had deliberately not been implemented for specific reasons, such as patient preferences. Another GP explicitly stated that he had wished for more “courageous” recommendations, which would have gone beyond the “evident” recommendations and would have challenged his previous prescribing decisions. GPs, however, also emphasized how the generation of only few recommendation for some of patients confirmed their prescribing decisions and work as physicians: “*I was happy, that the medication was not questioned in general. Otherwise, I would have had to doubt the quality of my work*” (GP, male, 44 years). The recommendations, or rather the lack thereof, was perceived as a confirmation of quality work by some GPs.

#### Implementation of prescribing recommendations

The implementation of prescribing recommendations generated by the STRIPA was one of the themes that was discussed during the interviews. In general, GPs confirmed the relatively low implementation rate with only a fraction of recommendations being implemented, which is in line with our first step’s quantitative findings. However, interviews showed differences between GPs in terms of how many recommendations they reported having implemented. Because the STRIPA sometimes did not capture all nuances of patient health status, GPs often had valid reasons to reject generated recommendations. Consequently, only a small percentage of recommendations was presented to and discussed with patients. One GP, however, also told us that while he was not able to implement many recommendations directly, seeing them with the tool helped him to become aware of potential prescribing problems. With regards to the implementation of recommendations that they deemed feasible, some GPs reported challenges when respect to presentation to patients. One GP expressed it like this: *“You have to be careful not to make yourself ‘lower’ than you are as a doctor. You should radiate a certain competence and not give the impression ‘I need a computer to help me treat you.’ Otherwise, it’ll be too complicated*” (male, 44 years).

Finally, the overall impressions of GPs were that the STRIPA was a potentially useful tool, but that its functionality was not ideal for regular use in clinical practice. For instance, a GP (male, 57 years) said, “*The STRIPA is actually very useful, even in the way in which it works right now, but it is too complex for everyday use.*” Another GP (male, 44 years) echoed this sentiment, “*If the STRIPA wants to get a chance, it has to run a lot smarter*,” meaning that data entry should be fully automated. Overall, while some GPs stated that their expectations were met, others stated that they were disappointed by the tool.

## Discussion

This mixed-methods study set out to explore the conduct of a medication review intervention centered around the use of the STRIPA in a real-life clinical setting during the OPTICA trial, a cluster-randomized controlled trial conducted in Swiss primary care settings. Our quantitative findings show that the expenditure of time for the preparation and use of the STRIPA as well as for the discussion of the recommendations generated was substantial, which may have limited the overall implementation of the intervention. Further, a small percentage of recommendations generated by the tool were presented to patients and implemented. The qualitative part of the study helped to explain the quantitative findings and showed that the main reasons for limited implementation of the STRIPA were related to problems with the data source, preparation of the eCDSS and its functionality, as well as the practicability of generated prescribing recommendations.

### Time factor

Both our quantitative and qualitative findings showed substantial time expenditures were required to prepare STRIPA, to run analyses and to discuss recommendations with patients. This finding is in line with the results from a process evaluation of a deprescribing intervention based on an eCDSS, in which GPs mainly reported retrieving additional information for the use of the tool to be time-consuming and inconvenient [32]. A previous study on the efficiency of medication reviews performed with the STRIPA showed that the time expenditure declined as professionals gained more experience (e.g., from around 20 to around 10 min per review) [43]. We unfortunately do not have any data to make comparisons about the time needed for medication review based on the STRIPA to other medication reviews performed by the same GPs in our sample.

### Data handling

Another major implementation challenge that we observed involved problems with data imports and the cumbersome nature of manual data entry, which was partially needed to add or update missing or incorrect information. In the OPTICA trial, the purpose of using data from electronic health records was to facilitate data entry for GPs. Despite this, most GPs reported that they had to spend a relatively large amount of time to manually update and add information as shown by the quantitative data (e.g., code diagnoses, update medication lists due to frequent changes in older multimorbid patients). In most cases, this was due to time lags following latest exports to the FIRE project database, which may have rendered an update necessary. There were also issues because not all data from the physicians’ electronic health record systems could be exported to FIRE (e.g., unstructured text information or certain lab values collected with different measurement units in different reference laboratories) and because different EHR systems exported data differently (e.g., reporting of medications and diagnoses at every encounter vs. reporting only when changes are made in the record). Some GPs criticized “missing information” in the data that had been imported into the STRIPA from their electronic health records programs via the FIRE project database. This may have resulted from GPs not knowing how data exported to the FIRE project were structured (i.e., that they were limited to selected values, or that data had to figure in the EHR system for a certain amount of time before inclusion in an export, which is why last-minute updates before an export may not have been captured).

### Implementation of prescribing recommendations

Another main barrier to the use of the STRIPA, which was shown by the quantitative findings and explained by the qualitative findings, was the relatively low implementation rate of recommendations generated by the tool. These findings are similar to previous ones from trials testing an eCDSS based on the STOPP/START criteria in hospital settings [23, 44, 45], one of which showed that 15% of all prescribing recommendations were implemented and the other one showed that 62% of patients had had ≥ 1 recommendation successfully implemented 2 months post-recommendation. Additionally, previous research on the usability of eCDSS-assisted de-prescribing found that 32% of GPs reported not having implemented any recommendation [33]. Interestingly, there seemed to be a wide variability between different GPs in previous studies. For instance, researchers found that while some GPs implemented nearly all generated recommendations, others implemented few or none [32]. While there is limited data about this in our study due to the small sample size, our findings suggest variability between GPs with regards to the implementation of prescribing recommendations (with the mean number of recommendations implementing ranging from 0.3 to 2.3). Furthermore, previous research has shown that more experienced healthcare professionals were more likely to disregard and reject recommendations [46]. Of note, a low implementation rate based upon generated recommendations is not necessarily bad; GPs may have had valid reasons for not implementing recommendations (e.g., recommendation not being appropriate for the patient, etc.), and it is not expected that every single prescribing recommendation should be implemented. A critical review of prescribing recommendations generated by eCDSS by clinicians is always required, as these tools can support clinicians but not replace their clinical judgment.

The reasons for implementation problems reported in the literature were similar to what we found in our qualitative analysis [32, 33]. First, the eCDSS did not capture all relevant patient-specific information, which is why some recommendations were not appropriate. This aligns with findings from the OPERAM trial, which had tested the STRIPA in hospital settings across four European countries and during which the medication review intervention was done during hospitalization [45]. Second, there were difficulties in implementing recommendations when prescribing decisions had been made by other medical specialists. Third, GPs’ or patients’ hesitancy toward medication changes can be major barriers to implementing recommendations. This is also reflected in the findings from the OPERAM trial, which found that the main reason for not implementing a recommendation was patients’ reluctance to change their medication use [45, 47]. These challenges need to be considered when further developing eCDSS. Despite the potentially low immediate implementation of recommendations, research shows that the use of eCDSS can be a useful tool to start reflections and discussions about patient medication use [48]. Hence, eCDSS-based interventions can positively influence GPs’ prescribing behaviors, as GPs have reported an increased awareness of prescribing problems after using a CDSS [33].

Even though some GPs reported a learning effect when performing the medication review using the STRIPA, we retrospectively assume that an average of 6 medication reviews may not have been enough to benefit from this learning effect. Performing such a small number of medication reviews using the STRIPA may not have allowed GPs to incorporate the use of the tool in their workflow in an efficient manner. Fragmented workflows are a commonly reported problem linked to the use of eCDSS, as these tools are often designed without considering the human information processing and behaviors [46]. While providing assistance to participating GPs during the study intervention, our study team noticed that the computer literacy differed between participating GPs. We assume that this influenced the STRIPA use during the trial. Consequently, working on better integrating the use of the STRIPA into the routine clinical practice of GPs and adapting it to computer literacy levels of individual GPs may be crucial for a successful implementation of eCDSS in primary care settings.

### Willingness to use eCDSS

Our findings showed that overall GPs would be willing to use eCDSS, such as the STRIPA, for medication review if the above-mentioned issues were addressed. This openness to using eCDSS is reflected in previous research [32]. In one study, 65% of respondents mentioned that they would be willing to use eCDSS in routine practice if the CDSS was integrated into their EHR system [33]. In addition to this, there would have to be minimal data entry so that the additional expenditure of time for using a tool would be as short as possible. Further, it is necessary that algorithms behind eCDSS must regularly be updated (e.g., with latest guidelines) [48]. Finally, our research clearly shows that simply providing new eCDSS to GPs is not sufficient and does not automatically translate into implementation of prescribing recommendations. GPs need to be supported with communication strategies on how to conduct shared decision-making with patients and strategies on how to overcome their own barriers to inappropriate prescribing.

Overall, qualitative findings suggest that GPs were dissatisfied with reoccurring problems when using the STRIPA (e.g., problems with data entry, generation of recommendations that GPs did not deem useful). Consequently, apart from solving technical issues and improving data imports, it will be crucial to work on presenting recommendations in a way that is perceived as useful by GPs. This is crucial, because instead of GPs focusing their energy on discarding non-useful recommendations, they should be able to focus on other potentially useful recommendations for prescribing decisions with older adults with multimorbidity and polypharmacy.

### Need for interoperable electronic health record systems in Swiss primary care settings

Direct, fully automated imports from the physicians’ EHR systems into the STRIPA would not have been technically feasible due to the multiple different EHR software providers used in the Swiss German language region of Switzerland. It thus made sense to collaborate with the FIRE project, as this was the best available option operationalizing EHR data for a clinical trial with an eCDSS in Switzerland. This mixed-methods study, however, shows this approach’s limitations. This should be a wake-up call for Swiss software developers to implement industrial standards allowing different EHR systems to be compatible with one another (e.g., feed data from one software into another, combine data from different software). In the future, this would allow easier use of eCDSS, such as the STRIPA. In addition, efforts should be made to make the coding of ICPC-2 diagnoses more common in Swiss primary care settings. At the moment, diagnostic coding is not commonly done in routine care, which affects the feasibility of implementing tools like the STRIPA.

Increasingly digitalized healthcare systems and readily available health data will allow the widespread use of eCDSS in the future. However, digitalization alone will not provide a sufficient basis for eCDSS to be used efficiently. Clinical practice and research must address the shortcomings identified in our research and in previous studies. In particular, approaches need to be developed to better integrate eCDSS into clinical workflows in primary care settings. Furthermore, EHR systems must become more interoperable for eCDSS to be effectively integrated into clinical workflows, so that data from different sources can be used reliably. If these challenges are successfully addressed, eCDSS can become a useful tool supporting physicians in primary care settings for optimizing prescribing practices.

### Strengths & limitations

The combined analyses of both quantitative and qualitative data allowed for better data triangulation and strengthened our findings. However, this mixed methods study has several limitations. First, since there were problems when generating PDF reports at the end of the STRIPA use, we had to retrospectively collect information on the prescribing recommendations by manually exporting them from the STRIPA. This came with the downside that we could only see which recommendations were generated, but not which ones had been accepted by GPs. This is why we had to rely on self-reported information from GPs regarding their acceptance of prescribing recommendations. Second, despite sending multiple reminders to GPs, we were faced with a small sample size and significant amount of missing quantitative data, as only 7 out of 21 GPs reported information about implementing prescribing recommendations, and only 8 out of 21 GPs agreed to be interviewed. Further, the sample of GPs mostly consisted of male GPs, which, in addition to the small sample size, could have limited the generalizability of findings. Next, we would like to acknowledge that the GPs who agreed to participate in the OPTICA trial and the qualitative interview were likely not representative of all GPs practicing in Swiss primary care settings. Finally, we did not consider patient perspectives on the conduct of the medication review intervention, which represents an important opportunity for future studies.

## Conclusion

Overall, GPs found the STRIPA useful, particularly due to its ability to generate recommendations based on large amounts of data. During the OPTICA trial, however, general practitioners only discussed and implemented a fraction of the recommendations generated by the STRIPA. Issues related to the STRIPA’s usability, general practitioners’ high expectations about the tool’s functionalities, data management, and time expenditure involved with preparing the STRIPA for analysis were important barriers described during semi-structured interviews. The qualitative findings help explain the low acceptance and implementation rate of the recommendations. Due to a learning effect, a decline in the expenditure of time needed to perform medication reviews with the STRIPA would be expected if GPs continued to use this tool more regularly and with more patients. In its current form, it is unlikely that the STRIPA will be implemented more broadly. Our results, however, are crucial for designing and adapting eCDSS like STRIPA in a meaningful way to make them more feasible and acceptable to providers and more suitable for regular use in primary care settings on a larger scale, as this will become increasingly possible in the context of digitalized healthcare systems.

### Electronic supplementary material

Below is the link to the electronic supplementary material.

Supplementary Material 1

## Data Availability

We will make the data for this study available to other researchers upon request. The data will be made available for scientific research purposes, after the proposed analysis plan has been approved. Data and documentation will be made available through a secure file exchange platform after approval of the proposal. In addition, a data transfer agreement must be signed (which defines obligations that the data requester must adhere to regarding privacy and data handling). Deidentified participant data limited to the data used for the proposed project will be made available, along with a data dictionary and annotated case report forms. For data access, please contact the corresponding author.

## Footnotes

1 Several GPs were male and 44 years old at the time of the interview.
