# Correction: A mixed methods analysis of the medication review intervention centered around the use of the ‘Systematic Tool to Reduce Inappropriate Prescribing’ Assistant (STRIPA) in Swiss primary care practices

**DOI:** 10.1186/s12913-024-10896-2

**Published:** 2024-04-02

**Authors:** Katharina Tabea Jungo, Michael J. Deml, Fabian Schalbetter, Jeanne Moor, Martin Feller, Renata Vidonscky Lüthold, Corlina Johanna Alida Huibers, Bastiaan Theodoor Gerard Marie Sallevelt, Michiel C. Meulendijk, Marco Spruit, Matthias Schwenkglenks, Nicolas Rodondi, Sven Streit

**Affiliations:** 1 Institute of Primary Health Care (BIHAM), University of Bern, Bern, Switzerland; 2 Institute of Sociological Research, University of Geneva, Geneva, Switzerland; 3 Department of General Internal Medicine, Inselspital, Bern University Hospital, University of Bern, Bern, Switzerland; 4 Geriatrics, Department of Geriatric Medicine, University Medical Center Utrecht, Utrecht University, Utrecht, The Netherlands; 5 Department of Clinical Pharmacy, University Medical Center Utrecht, Utrecht, Utrecht, The Netherlands; 6 Public Health and Primary Care (PHEG), Leiden University Medical Center, Leiden University, Leiden, Netherlands; 7 Leiden Institute of Advanced Computer Science (LIACS), Faculty of Science, Leiden University, Leiden, Netherlands; 8 Department of Information and Computing Sciences, Utrecht University, Utrecht, Netherlands; 9 Health Economics Facility, Department of Public Health, University of Basel, Basel, Switzerland; 10 Institute of Pharmaceutical Medicine (ECPM), University of Basel, Basel, Switzerland; 11 Epidemiology, Biostatistics and Prevention Institute (EBPI), University of Zurich, Zurich, Switzerland; 12 School for Health Sciences, University of Bern, Bern, Switzerland; 13 Center for Healthcare Delivery Sciences (C4HDS), Division of Pharmacoepidemiology and Pharmacoeconomics, Department of Medicine, Brigham and Women’s Hospital and Harvard Medical School, Boston, USA

**Correction to:** ***BMC Health Services Research*** **(2024) 24: 350**

10.1186/s12913-024-10773-y

In this article, the author name Corlina Johanna Alida Huibers was incorrectly written as Johanna Alida Corlina Huibers due to a typesetting mistake. The author group has been updated above and the original article has been corrected. The publisher apologises to the authors and readers for the inconvenience caused by this error.

