# Supplementary material for: A mixed methods analysis of the medication review intervention centered around the use of the ‘Systematic Tool to Reduce Inappropriate Prescribing’ Assistant (STRIPA) in Swiss primary care practices
Source: BMC Health Serv Res. 2024 Mar 18;24:350. doi: 10.1186/s12913-024-10773-y (PMC10949561; doi:10.1186/s12913-024-10773-y)
Supplement: Supplementary file 1 — Supplementary Material 1 [file 12913_2024_10773_MOESM1_ESM.docx]

**A Mixed Methods Analysis of the Medication Review Intervention Centered Around the Use of the ‘Systematic Tool to Reduce Inappropriate Prescribing’ (STRIP) Assistant in Swiss Primary Care Practices**

Katharina Tabea Jungo^1^, Michael J. Deml^2^, Fabian Schalbetter^1^, Jeanne Moor^1,3^, Martin Feller^1^, Renata Lüthold^1^, Corlina Johanna Alida Huibers^4^, Bastiaan Theodoor Gerard Marie Sallevelt^5^, Michiel C Meulendijk^6^, Marco Spruit^6,7,8^, Matthias Schwenkglenks^9,10^, Nicolas Rodondi^1,3^, Sven Streit^1^

***Affiliations***

^1^ Institute of Primary Health Care (BIHAM), University of Bern, Bern, Switzerland

^2^ Institute of Sociological Research, University of Geneva, Geneva, Switzerland.

^3^ Department of General Internal Medicine, Inselspital, Bern University Hospital, University of Bern, Bern, Switzerland.

^4^ Geriatrics, Department of Geriatric Medicine, University Medical Center Utrecht, Utrecht University, Utrecht, The Netherlands.

^5^ Department of Clinical Pharmacy, University Medical Center Utrecht, Utrecht, Utrecht, The Netherlands.

^6^ Public Health and Primary Care (PHEG), Leiden University Medical Center, Leiden University, Leiden, Netherlands.

^7^ Leiden Institute of Advanced Computer Science (LIACS), Faculty of Science, Leiden University, Leiden, Netherlands.

^8^ Department of Information and Computing Sciences, Utrecht University, Utrecht, Netherlands.

^9^ Health Economics Facility, Department of Public Health, University of Basel, Basel, Switzerland.

^10^ Epidemiology, Biostatistics and Prevention Institute (EBPI), University of Zurich, Zurich, Switzerland.

**Interview Guide *(translated from German)***

**Introductory questions**

*Usual care of older adults with multimorbidity and polypharmacy*

- How often per week do you see elderly patients over the age of 65 with polypharmacy, i.e., 5 or more medications taken regularly, and multimorbidity, i.e., 3 or more chronic conditions, in your clinical practice?
  - Probes:
    - What percentage of your patients do you estimate this patient group makes up?
    - How regularly do you see such patients?
- Can you describe these patients?
  - Probes:
    - Can you name any examples of their characteristics?
    - And how they compare to other patients?
- Can you describe a typical consultation with such a patient?
  - Probes:
    - Can you give an example?
    - Do you use any tools to help you treat these patients and can you describe how you use them?
    - Do you encounter any difficulties when treating these patients? Do you have any examples of this?

*Medication optimization*

- How do you typically approach medication changes in your multimorbid patients with polypharmacy?
- How would you describe your willingness to change medications in this patient population?
  - Probes *(for both questions above):*
    - What helps you optimize medications in this patient group?
    - What obstacles do you encounter in this process?
    - What is the patient's role in this process?

*Role of the general practitioner*

- How do you see your own role as a general practitioner in the care of such a patient with multimorbidity and polypharmacy?
  - Probe:
    - What do you find most difficult about your role?
- How could you, as a general practitioner, be better supported in the care of this patient population?
  - Probes:
    - Do you have a specific idea or suggestion for improvement?
    - How could your electronic medical record system be adapted to support you in treating this patient population and optimize their medication?

**STRIP Assistant intervention**

*Experience with similar tools*

- Do you already know of tools that are similar to the STRIP Assistant?
  - Probes:
    - If yes, which ones?
      - Have you already used them in your clinical practice?
      - What problems or positive factors did you encounter when using these tools?
      - How were these tools different from the STRIP Assistant?
- Can you describe for me how the STRIP Assistant works?
  - Probes:
    - What do you know about the STOP/START criteria?

*Preparation of the intervention*

- Did you use the STRIP Assistant informational materials, namely the video and written instructions?
  - If so, how much time did this take?
- Can you elaborate on your impression of the STRIP Assistant informational materials (video and written instructions)?
- *We saw in the REDCap study database that it took 40 minutes per patient to prepare and perform the intervention.* How does that compare to your experience during the trial when conducting the intervention?
  - Probe:
    - Can you explain why it may have taken some general practitioners 15 minutes and others up to 60 minutes?

*Use of the STRIP Assistant*

- *We saw that generally 23% of STRIPA recommendations were presented to patients, 7% were not presented to patients, and for 70% we don't know.^[[1]](#footnote-1)^* Does this information surprise you?
  - Why yes/why not?
  - Probes:
    - To what extent were the recommendations relevant to your patients?
    - How would you rate the quality of the STRIPA recommendations that were generated for your patients?
- *We found in our data that 13% of the recommendations presented were ultimately implemented. 10% of those recommendations were not implemented. For 77%, we don't know.* Does this number surprise you?
  - - Why yes/why not?
  - Probes:
    - How were the recommendation from the STRIP assistant received by your patients?
    - *(Only if any recommendations were implemented by GP/interview partner)* *We saw that shared decision making between primary care physician(s) took an average of 8 minutes.* To what extent were you able to conduct shared decision making with your patients?
      - Probes:
        - What problems did you encounter in this process?
        - What facilitated this decision making process?
- To what extent were your expectations of the STRIP assistant met?
  - Probes:
    - Has the STRIP Assistant changed the way you treated older multimorbid patients with polypharmacy?
      - If yes, to what extent?
    - Can you describe for me what aspects you liked and disliked about using the STRIP Assistant?
      - Probes:
        - Where is there room for improvement?
        - Would you continue to use the STRIP software in your practice?

Why yes/why no?

- In which step of using the STRIP Assistant do you see the greatest difficulties?
- And at which step do you see the greatest potential?

**Implementation of electronic clinical decision support systems**

*Advantages and disadvantages*

- What advantages do you see in using STRIP or a similar electronic decision support system?
- What do you think are the disadvantages?

*Use of electronic clinical decision support systems in everyday clinical practice*

- Under what conditions would you be willing to integrate the use of such an electronic decision support system into your everyday clinical practice?
  - Probe:
    - Why yes/ why not?
    - Would you be willing to pay an annual price for the use of the STRIP Assistant?
- In your opinion, to what extent should such electronic clinical decision support systems be integrated into electronic medical record systems?
- In your opinion, what impact can the use of an electronic decision support systems have on the daily practice of general practitioners?

*Impact on patient outcomes*

- To what extent do you think the use of electronic decision support systems, such as the STRIP Assistant, can have an impact on patients?
  - Probe:
    - Which patient outcomes would likely be most affected?

*Future of electronic decision support systems in Swiss primary care settings*

- How do you see the future of such of electronic decision support systems in Swiss primary care settings?
- How would you go about implementing the STRIP Assistant or similar electronic decision support systems in Swiss primary care settings at a larger scale?
  - Do you have any other comments about implementing electronic decision support systems in Swiss primary care systems?

**Conclusion**

- Is there anything we have already discussed but you would like to clarify?
- Are there any outstanding suggestions or comments that you would like to share?
- Is there anything we have not yet discussed but you find relevant to this topic and would like to share?

1. Preliminary data were used to inform the interview guide, which is why the numbers are slightly different from the quantitative findings presented in the paper. [↑](#footnote-ref-1)
